# Influence of Hot Chlorinated Water and Stabilizer Package on the Fatigue Crack Growth Resistance of Glass Fiber Reinforced Polyamide

**DOI:** 10.3390/polym10080829

**Published:** 2018-07-27

**Authors:** Joerg Fischer, Patrick R. Bradler, Mohamad H. Akhras, Gernot M. Wallner, Reinhold W. Lang

**Affiliations:** Johannes Kepler University Linz, Institute of Polymeric Materials and Testing, Altenberger Strasse 69, 4040 Linz, Austria; patrick.bradler@jku.at (P.R.B.); solpol@jku.at (M.H.A.); gernot.wallner@jku.at (G.M.W.); reinhold.lang@jku.at (R.W.L.)

**Keywords:** fatigue crack growth resistance, glass fiber reinforced polyamide, superimposed mechanical-environmental testing, chlorinated water, elevated temperature, stabilizer system

## Abstract

To assess the potential use of polyamide (PA) for solar-thermal systems applications, the effect of water with varying chlorine content on the fatigue crack growth (FCG) resistance of two PA formulations differing in their stabilizer packages was investigated at 80 °C. A commercial PA containing 30 wt % glass fibers and a standard stabilization package (PA-0) was used as the reference material. For the other formulation, the reference material PA-0 was compounded with two additional stabilizers (PA-S1). Keeping the specimen geometry and initial loading conditions the same, the total number of cycles to ultimate specimen failure was found to be reduced with an increase in chlorine content for both materials. As to the effect of the chlorine content on crack growth kinetics, the most pronounced effect in enhancing the crack growth rates or decreasing the FCG resistance was determined between 0 ppm and 1 ppm chlorine content. When comparing the relative change of FCG resistance in chlorinated water (10 ppm) to the FCG resistance in non-chlorinated water (0 ppm), the additional stabilization in the material PA-S1 appears beneficial over the stabilization in the reference material PA-0.

## 1. Introduction

Solar-thermal technologies are typically categorized into pumped (active) and non-pumped (passive) solar-thermal systems, and they are mainly utilized for domestic hot water and solar assisted space heating [1]. Over the last two decades, many efforts have been made to improve solar-thermal systems in terms of costs, weight, design, and overall performance by replacing conventional materials like copper, aluminum or stainless steel with polymeric materials [2]. However, numerous system components are exposed to high and alternating mechanical stresses superimposed by various environments (e.g., air, liquids, and elevated temperatures), which may pose a significant challenge to plastics.

Glass fiber reinforced polyamides (PA-GF) offer a high potential for applications where low costs, a high strength-to-weight ratio, thermal and chemical stability, as well as freedom in design is demanded [3,4,5]. Based on their excellent property profile, PA-GF grades are applied in numerous rather demanding parts in the automotive industry, and they have been revealed to be a reliable material for these applications [3]. As to solar-thermal systems, PA-GF is also utilized in the endcaps of steel pipes in integrated storage collectors [6,7], in push fittings for pipes, and as a material for mounting [7,8,9].

Most interestingly, in numerous recent and current research projects [1,7,8,10], PA-GF grades are also considered for developing large pressurized solar-thermal absorbers and storage tanks, which are exposed to various environmental media. Usually, their exterior is surrounded by air and a heat carrier fluid is flowing inside. The fluid is dependent on the collector type and the climatic conditions [2]. In non-pumped, single-loop solar-thermal systems such as in an integrated storage collector, for which good market perspectives exist, the heat carrier fluid typically is potable water. However, as a prevention of waterborne diseases, disinfection of water contaminated with waterborne pathogens is required [11]. Chlorine, based on an aqueous solution of chlorine gas or sodium hypochlorite, is the most affordable and most utilized water disinfectant [11,12,13]. As chlorine contents in drinking water of more than 5 ppm (mg/L) may lead to intense odor and taste or even to health risks, the World Health Organization (WHO) set the maximum value of free chlorine in drinking water to 5 ppm [11]. Nevertheless, due to the increasing oxidation-reduction potential with higher chlorine contents, for some applications higher chlorine contents than the 5 ppm suggested by the WHO are utilized. Simultaneously, the pH value of disinfected drinking water should be between 6.5 and 7.6. In this pH range, the higher reactive free chlorine species hypochlorous acid is predominant [14,15].

Due to the hydrophilic behavior of polyamides (PAs), depending on the PA grade, a moisture absorbance of up to 8 wt % may occur, with the moisture uptake primarily taking place in the amorphous phase, resulting in the well reported phenomenon of moisture plasticization, which acts to reduce the glass transition temperature. Acting as a plasticization agent, any moisture uptake profoundly affects the mechanical property profile, and at elevated temperatures irreversible hydrolytic decomposition and deterioration of mechanical properties may also take place [5,8,16,17,18,19,20,21,22]. Moreover, based on the high oxidation-reduction potential of chlorine, polyamide is very sensitive to chlorinated water [23,24,25,26,27,28]. In the literature [26], a strong interdependence between chlorine sensitivity and molecular structure of polyamide is reported. While aromatic polyamides are very sensitive to chlorine-induced oxidation, aliphatic and cyclo-aliphatic polyamides are more resistant. Of high practical significance, the use of chlorine often leads to a pronounced reduction of the mechanical performance of polyamides [24]. Strong evidence also exists that the simultaneous exposure of polymeric materials in general to high mechanical stresses and degrading environments leads to an accelerated deterioration of properties, particularly under long-term loading situations [7,8,10,15,17,29,30,31,32,33,34,35,36,37,38]. Hence, any profound laboratory assessment of the performance potential of plastics under superimposed mechanical and environmental service loading conditions, as is clearly the case for solar-thermal components, must account for these simultaneous influences.

Considering the potential application of PA-GF materials in advanced solar-thermal applications, for which intermittent or cyclic loading is quite typical (e.g., day-night cycles, service-induced cycles, etc.), the objective of this research was to investigate the effect of hot water with varying chlorine contents on the fatigue crack growth (FCG) resistance of such materials. For example, in single-loop, integrated storage collectors with temperature dependent internal pressures ranging from 4 bar to 6 bar [6], mostly disinfected potable water is used as heat carrier fluid, and in Southern European countries a maximum stagnation temperature of about 80 °C is attained [18]. Thus, in this research, the failure behavior of two PA-GF formulations differing in their stabilizer packages was investigated in water with varying chlorine contents at 80 °C.

## 2. Materials and Methods

### 2.1. Materials

A commercial PA-GF containing 30 wt % glass fibers and a standard heat stabilization package, designated in this study as PA-0, was used as the reference material. For the other formulation, designated as PA-S1, the reference material PA-0 was compounded with a PA masterbatch containing two additional stabilizers for processing and long-term performance.

The commercial reference material PA-0 was of the type Zytel 70G30HSL NC010 (Du Pont), which is a PA66 grade reinforced with 30 wt % of short glass fibers. It is a heat stabilized injection-molding grade. For PA-S1, the additional stabilizer package was added via injection molding of PA-0 together with a pre-compounded masterbatch, using an injection-molding machine of the type Victory 60 (Engel Austria GmbH, Schwertberg, Austria). The masterbatch contained 90 wt % supporting material, which acts as matrix material. As supporting material, the PA 6 grade DOMAMID AQ 500 (Domo Chemicals, Leuna, Germany) was used. For the additional 10 wt % of the masterbatch, the stabilizer package consisted of the two stabilizers Irganox 1098 (BASF AG, Ludwigshafen, Germany) and Stabilizer 9000 (Raschig GmbH, Ludwigshafen, Germany). Irganox 1098 is a phenolic primary antioxidant and Stabilizer 9000 is a polymeric carbodiimide for stabilization against hydrolysis. Furthermore, polymeric carbodiimide reacts with carboxylic acid end groups resulting in chain extension or cross-linking [39,40]. An overview of the composition of the two material formulations investigated is provided in Table 1. It should be noted, that for the formulation of PA-S1 with 90 wt % of the glass fiber reinforced PA-0 plus 10 wt % of a non-glass fiber reinforced masterbatch, an overall glass fiber content of 27 wt % results compared to the glass fiber content of 30 wt % for PA-0. In a dry state, both material formulations have a glass transition temperature of about 60 °C and a melting temperature of about 260 °C. After pre-conditioning (water saturation), the glass transition temperatures decreased due to water uptake to about −20 °C.

### 2.2. Specimens

**Figure 1:**
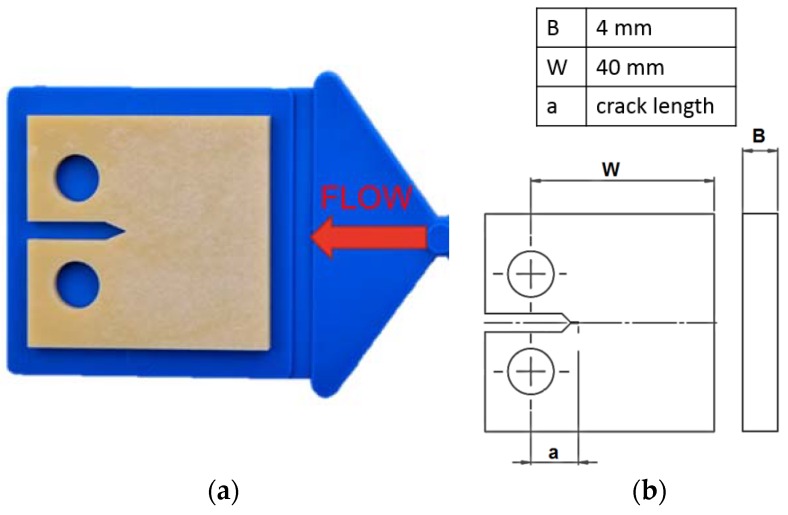
(**a**) Injection molded plaque (blue) and positioning of compact type (CT) specimen with the notch in melt-flow direction; (**b**) CT specimen with dimensions.

The FCG tests were conducted with compact type (CT) specimens, which were milled of injection molded plaques with the dimensions of 60 mm × 60 mm × 4 mm. As the fiber orientation in short-glass fiber reinforced plastics affects the FCG resistance [7,41], the CT specimens were milled out with notches in injection melt-flow direction as shown Figure 1a. Figure 1b provides information on the geometry and dimensions of the CT specimen. To avoid geometrical imperfections, the CT specimens were milled with a 4-axis milling machine EMCO Mill E600 (EMCO Group, Hertford, UK). An initial crack was cut at the notch tip of the CT specimen with a razor blade by broaching. For this purpose, a universal testing machine Z2.5 (Zwick Roell, Ulm, Germany) together with a special designed clamp for the razor blade was used.

### 2.3. Fatigue Crack Growth Tests

**Figure 2:**
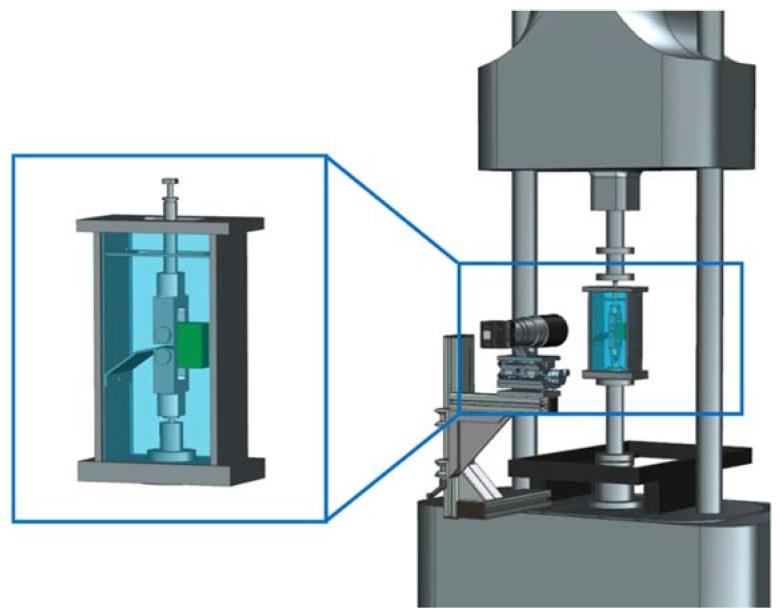
Schematic illustration of the test system for superimposed mechanical-environmental loading, also providing details on the design of the environmental containment. The test system is equipped with an in situ optical crack length measurement device.

All cyclic tests were carried out on an electro-dynamic testing machine of the type ElectroPuls E3000 (Instron, Norwood, MA, USA). The testing machine was equipped with a crack length measurement device containing a 12 megapixel, APS-C size sensor LXG-120M camera (Baumer Holding AG, Frauenfeld, Switzerland) and a Micro-Nikkor AF 200 mm f/4 D ED lens (Nikon Corporation, Tokyo, Japan) directly mounted to the machine frame (see Figure 2) [42]. A computer program processed the captured images [43]. For the crack length measurements, the image pixels were compared in terms of grey scales. As a result, the program allocates the crack length together with the corresponding cycle number.

Additionally, a chlorinated water test arrangement was used allowing in situ tests with the intended chlorine contents, pH, and temperature [15]. Specimens were loaded with a sinusoidal force profile with a frequency of 5 Hz in non-chlorinated water and 10 Hz in chlorinated water, respectively, and an R-ratio (ratio between minimum and maximum applied force F) of 0.1. This frequency adaptation from 5 Hz to 10 Hz was needed to ensure that the total test time of chlorinated experiments remained within a maximum of 24 h. It should be emphasized, that a difference in the test frequency from 5 Hz to 10 Hz was found to be negligible in affecting the FCG behavior in glass fiber reinforced polyamides [44]. All tests were conducted at 80 °C. In the chlorinated water experiments, the chlorine content was set to 1 ppm, 5 ppm, and 10 ppm, and a pH of 7. In addition, non-chlorinated water experiments were conducted (i.e., 0 ppm free chlorine). For each of the materials, the mechanical loads in the fatigue experiments in chlorinated water were kept constant for all chlorine concentrations with a maximum force in the sinusoidal loading cycle of 330 N and 300 N for PA-0 and PA-S1, respectively. The applied maximum forces were selected on the one hand to ensure quasi-brittle crack growth over a sufficient wide regime of crack growth rates, and to simultaneously keep the total testing time of the FCG experiment within a maximum of 24 h. The slightly increased force range for PA-0 compared to PA-S1 thus accounts for the higher overall glass fiber content in the former formulation, which causes a somewhat higher FCG resistance [45,46,47].

Prior to fatigue testing, all specimens were pre-conditioned in the specific test liquid at 80 °C for 7 days to ensure saturation level of media uptake. To compensate for the loss in chlorine content at elevated temperatures, the chlorinated water was renewed every 12 h. The weight of all specimens was recorded prior and after the media exposure to saturation, and for the chlorine contents of 0 ppm, 5 ppm, and 10 ppm the weight change of the specimens was also recorded in regular intervals at intermediate stages of media exposure.

### 2.4. Data Reduction

The stress intensity factor range values ∆*K_I_* for CT specimens [48] were calculated according to Equations (1) and (2), where “∆*F*” represents the applied sinusoidal force range and “*a*” the crack length. The parameters “*B*” and “*W*” correspond to the thickness and width of the specimen, respectively (Figure 1b).

(1) $$\Delta K_{I}=\frac{\Delta F}{B\times \sqrt{W}}\times f\left(\frac{a}{W}\right)$$

(2) $$f\left(\frac{a}{W}\right)=\frac{\left(2+\frac{a}{W}\right)}{{\left(1-\frac{a}{W}\right)}^{3/2}}\times \left(0.886+4.64\times \left(\frac{a}{W}\right)-13.32\times {\left(\frac{a}{W}\right)}^{2}+14.72\times {\left(\frac{a}{W}\right)}^{3}-5.6\times {\left(\frac{a}{W}\right)}^{4}\right)$$

**Figure 3:**
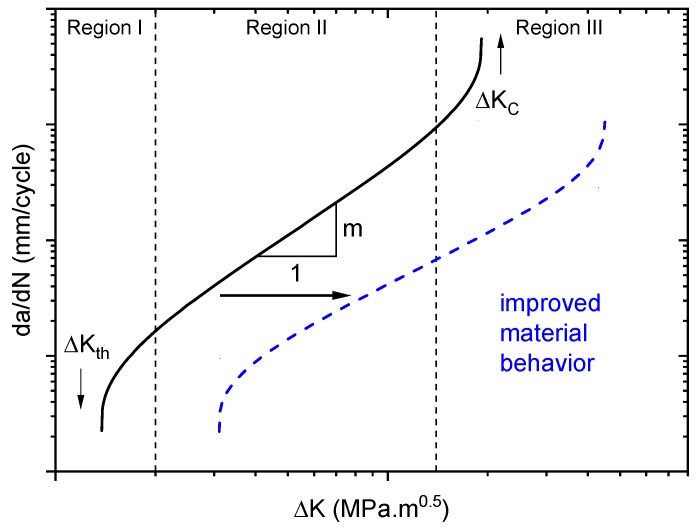
Schematic plot of the crack growth rate *da*/*dN* as a function of the stress intensity factor range ∆*K*.

For each testing condition, fatigue tests were repeated twice and equivalent results with only minor deviations were obtained (∆*K_I_* deviations for a pre-defined test speed remained below 5%). This reflects the rather high reproducibility of fatigue crack growth (FCG) experiments in general, so that two test runs were considered sufficient in terms of reproducibility. Results were plotted in terms of log *da*/*dN* vs. log ∆*K*. Frequently, in fatigue crack growth curves, three different regions may be distinguished when covering a sufficiently wide range of crack growth rates (see Figure 3). Region I describes the fatigue crack growth threshold regime, where crack growth rates become vanishingly small as ∆*K* is decreased. Region II corresponds to the stable crack growth regime, and Region III to the regime of unstable crack growth. Stable crack growth in Region II is typically described by the Paris relationship presented in Equation (3), where “*A*” and “*m*” are parameters depending on material and test conditions.

(3) $$\frac{da}{dN}=A\times \Delta K_{I}{}^{m}$$

When comparing different materials or test conditions, improved fatigue crack growth resistance is reflected by a shift of the FCG curve to lower crack growth rates or to higher ∆*K* levels, respectively [49]. Moreover, a lower number for the exponent “*m*” in Equation (3) corresponding to a lower slope in Region II of Figure 3 is generally considered beneficial.

## 3. Results

### 3.1. Specimen Pre-Conditioning and Water Absorption

**Figure 4:**
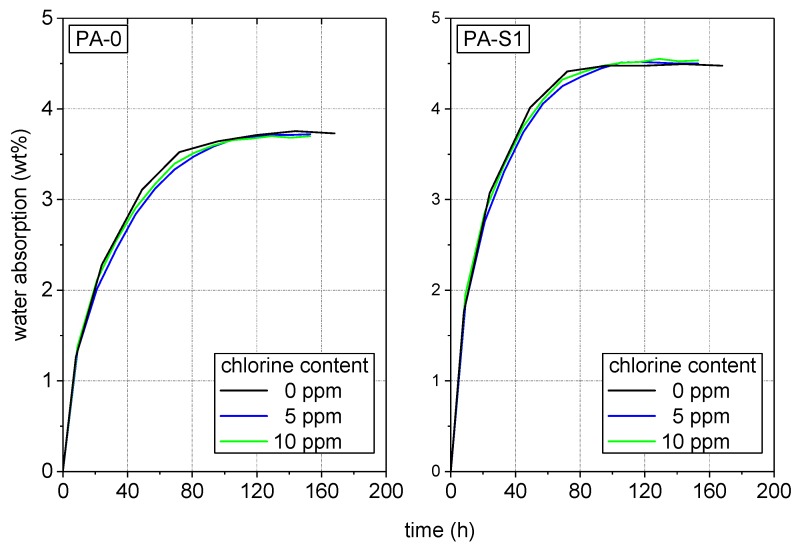
Time dependent water absorption of PA-0 and PA-S1 pre-conditioned in non-chlorinated water and chlorinated water with different chlorine contents.

Reflecting the range of service conditions of solar-thermal system components, specimens of each of the two materials were first immersed in non-chlorinated water (0 ppm free chlorine) and in chlorinated water with different chlorine content (5 ppm and 10 ppm free chlorine) to water absorption saturation at 80 °C prior to testing. Figure 4 depicts the time-dependent water absorption for the two PA formulations under these conditions. Most importantly, the water absorption in both materials apparently is independent of the chlorine content. While a saturation water level of 3.7% was reached for PA-0 after about 120 h, for PA-S1 the saturation water uptake amounted to 4.5% and was reached after about 100 h. The somewhat higher water absorption and the shorter time to reach the saturation level for PA-S1 is consistent with the slightly lower glass fiber content of this material (Section 2.1). Moreover, the reported higher tendency of PA6, the base polymer of the masterbatch used for the PA-S1 compound, to absorb moisture compared to PA66 may have also contributed to the higher total water absorption of PA-S1 [5,22,50].

**Figure 5:**
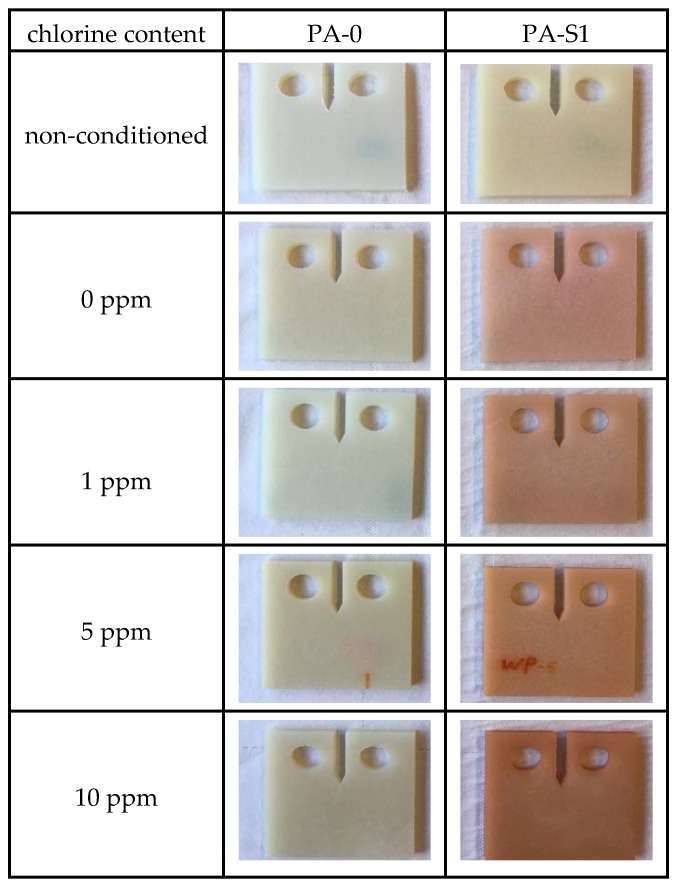
Specimen appearance affected by pre-conditioning of specimens in non-chlorinated and chlorinated water with different chlorine contents for 7 days at 80 °C.

In Figure 5, images of the specimens after pre-conditioning to the saturation level in the various environments are compared to the non-conditioned material state. Clearly, for both materials the chlorine content affected the optical appearance of the specimens. However, while specimens of PA-0 revealed a tendency towards a darker appearance without any significant coloration, specimens of PA-S1, when moisturized in non-chlorinated and chlorinated water exhibited some reddish coloration, which became darker with increasing chlorine content. Apparently, this reddening was caused by the interaction of water with the additional stabilizer package in PA-S1, and thus was related to the presence of either the phenolic primary antioxidant Irganox 1098 or the polymeric carbodiimide Stabilizer 9000 or even the combination of both stabilizers.

### 3.2. Fatigue Crack Growth Resistance

In the next subsection, the effect of chlorine content on the FCG behavior under superimposed environmental-mechanical loading conditions will first be discussed separately for both materials investigated. In a subsequent subsection, the effect of the added stabilizer system in PA-S1 will be compared to the reference material PA-0.

#### 3.2.1. Effect of Chlorine Content

**Figure 6:**
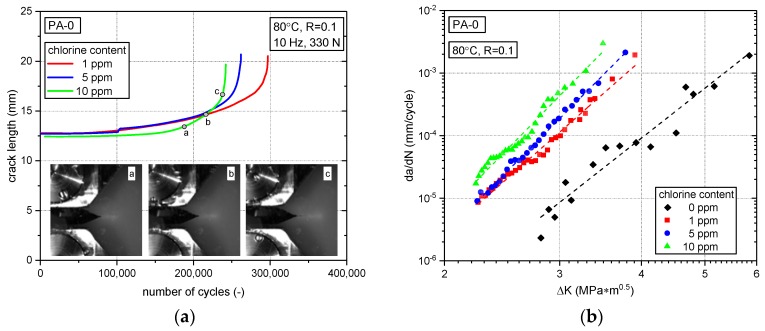
Fatigue crack growth of PA-0 in various environments. (**a**) Crack length vs. number of cycle curves for experiments with specimens of identical geometry, initial crack length and loading under various chlorinated environments along with representative specimen side-surface images showing the crack extension for a test performed with 10 ppm free chlorine; (**b**) Fatigue crack growth curves in non-chlorinated water and in chlorinated water with different chlorine contents.

Figure 6 illustrates the results of the FCG experiments performed with PA-0 under the various liquid environmental test conditions. To start with, the crack propagation as measured in terms of crack length vs. number of loading cycles is shown in Figure 6a for specimens tested in chlorinated water. The crack advance for tests in 10 ppm chlorinated water is indicated by dots for three crack length positions a, b, and c The corresponding images of the respective crack tip regions are also inserted in Figure 6a. All specimens tested in chlorinated water were of the same geometry including an identical initial crack length of about 12.5 mm when the test was started. Figure 6a also shows the number of cycles to ultimate specimen failure for each of the test conditions (end point in each of the crack length vs. number of cycle curves, where the crack extension becomes unstable). Clearly, the number of cycles to failure decreases with increasing chlorine content. For comparison, specimens of the identical specimen geometry, however, with a slightly longer initial crack length of about 14 mm but same cyclic load range (i.e., maximum force of 330 N) sustained up to ca. 700,000 cycles to ultimate failure. It should be noted, that this is more than twice the number of cycles to failure observed for chlorinated water, despite the fact that a somewhat higher initial stress intensity factor range was applied in non-chlorinated water.

A more detailed picture of the kinetics of crack growth is provided in Figure 6b, where crack growth rates *da*/*dN* are plotted vs. the stress intensity factor range ∆*K*. Here, the data under chlorinated conditions are also compared to experimental results in non-chlorinated water. While essentially over the entire fatigue crack growth range investigated a higher chlorine content leads to increasing crack growth rates, the most significant effect was to be seen between 0 ppm and 1 ppm chlorine content. As the chlorine content was further increased from 1 ppm to 10 ppm, the deteriorating effect of an enhanced chlorine content relatively diminished.

**Figure 7:**
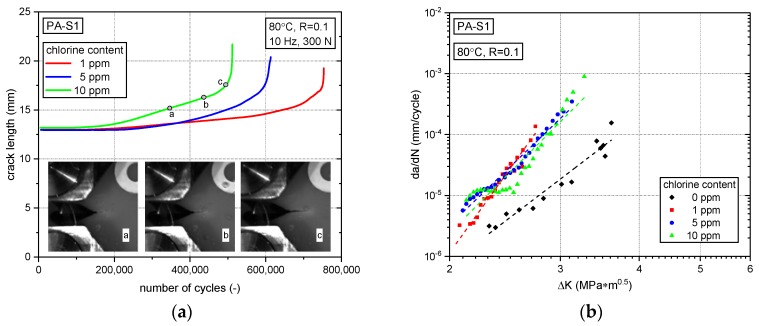
Fatigue crack growth of PA-S1 in various environments. (**a**) Crack length vs. number of cycle curves for experiments with specimens of identical geometry, initial crack length and loading under various chlorinated environments along with representative specimen side-surface images showing the crack extension for a test performed with 10 ppm free chlorine; (**b**) Fatigue crack growth curves in non-chlorinated water and in chlorinated water with different chlorine contents.

Analogous experimental findings and results are shown in Figure 7 for PA-S1. Again, for specimens of identical specimen geometry and initial crack length as well as equivalent cyclic loads the total number of cycles for ultimate specimen failure was found to decrease markedly from 1 ppm to 10 ppm chlorine content (see Figure 7a). Moreover, for identical test conditions, as was the case with PA-0 in Figure 6a, the number of cycles to ultimate failure for non-chlorinated test conditions was again found to be about 1.6 to 2.7 times as high as for chlorinated test conditions (ca. 1,200,000 cycles in non-chlorinated water vs. less than ca. 750,000 in chlorinated water).

When comparing the results of experiments in the various chlorinated water environments with the non-chlorinated water environment in terms of FCG kinetics (see Figure 7b), the most pronounced detrimental effect on the FCG resistance of PA-S1 appears again between 0 ppm and 1 ppm free chlorine. However, any further increase in the chlorine content from 1 ppm to 10 ppm does not show such a clear tendency of the chlorine concentration on FCG rates as was the case for PA-0. In fact, the FCG curves seem to cross over somewhat, and the clear ranking on specimen lifetime when measuring the total number of cycles to ultimate specimen failure (see Figure 7a) may perhaps be related to different contributions of the crack growth initiation stage vs. the stable crack growth stage in the different chlorinated water environments. 

#### 3.2.2. Effect of Stabilizer System

**Figure 8:**
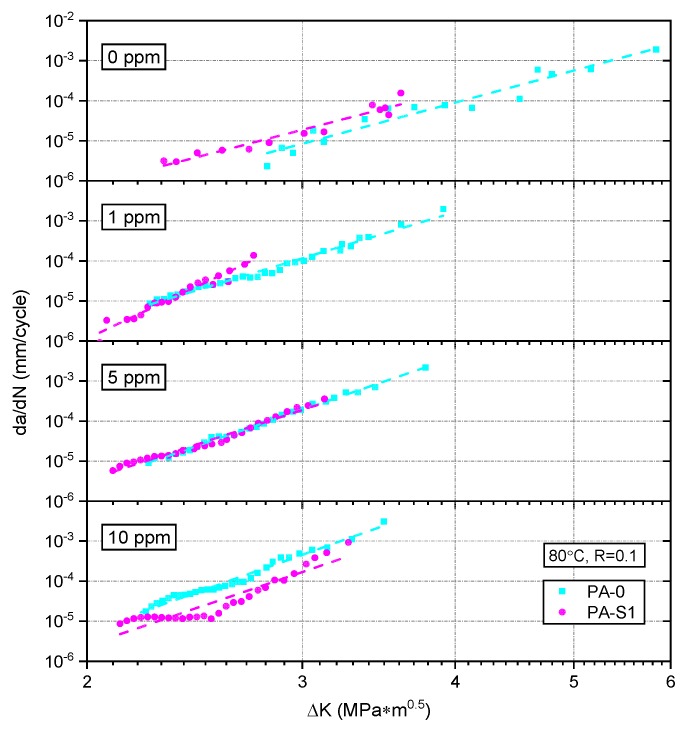
Comparison of the fatigue crack growth curves of PA-0 and PA-S1 tested in non-chlorinated water and chlorinated water with different chlorine contents.

In Figure 8 the FCG curves are illustrated in terms of the effect of stabilizer system. Therefore, a direct comparison of the FCG curves for the PA grades PA-0 and PA-S1 are depicted for the different chlorine contents 0 ppm, 1 ppm, 5 ppm, and 10 ppm. While for the chlorine contents of 1 ppm and 5 ppm the FCG curves of the three PA grades were close together with only slight differences in slope, for 0 ppm and 10 ppm clear distinction between the FCG behavior of both formulations is possible. For 0 ppm free chlorine an inferior FCG resistance of PA-S1 was obtained. However, for 10 ppm a significantly improved FCG resistance was determined for PA-S1. Hence, the two additional stabilizers Irganox 1098 and Stabilizer 9000 led to a superior FCG behavior at higher chlorine contents.

When comparing the FCG curve of PA-0 and PA-S1 under identical environmental conditions, as is depicted in Figure 8, an interesting observation was made. In non-chlorinated water (0 ppm), the FCG curve of PA-0 shifted to higher ∆*K* levels compared to PA-S1, thus indicating a higher FCG resistance of PA-0. This most likely reflects the higher short-glass fiber content of PA-0, a result which is in good agreement with findings by others [8,10,45,46,47,51,52]. The higher FCG resistance with increasing fiber content is related to (1) the stress transfer from the matrix to the much stronger fibers along with the overall increase in specimen stiffness; (2) the additional energy dissipation mechanisms associated with debonding, fiber breakage and fiber pull-out, and local plastic deformation in the matrix around the fibers; and (3) crack blunting due to the more complex damage zone [41,51]. Conversely, the FCG ranking of the two material formulations changed when testing the materials in chlorinated water with 10 ppm free chlorine. Under these conditions, PA-S1 exhibited an improved FCG resistance over PA-0, thus clearly corroborating the beneficial influence of the added stabilizer package in PA-S1. At intermediate chlorine concentrations (1 ppm and 5 ppm), the two materials seem to show nearly equivalent FCG behavior, indicating that the effects of the higher glass fiber content in PA-0 and the added stabilizer package in PA-S1 balanced each other out.

**Figure 9:**
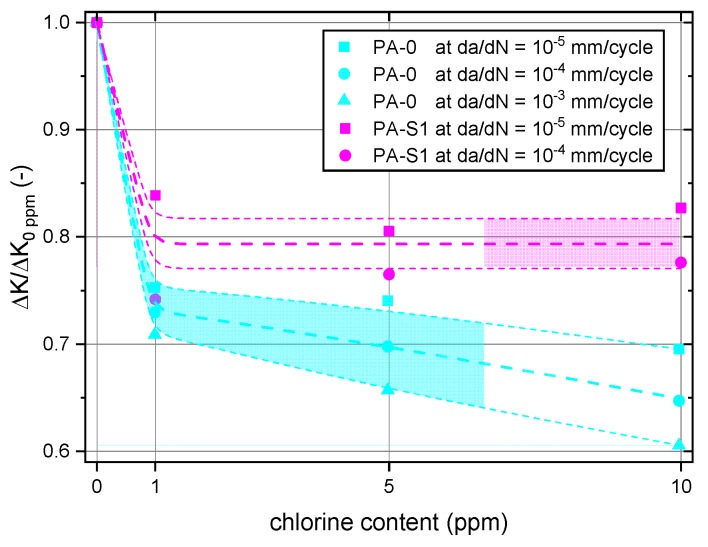
Effect of chlorine content on the normalized stress intensity factor range ∆*K*/∆*K*_0 ppm_ (*da*/*dN* = constant) for PA-0 and PA-S1.

To better visualize the influence of the additional stabilizer system on the FCG resistance in the different liquid media, the FCG results for both material formulations are depicted in a normalized manner in Figure 9. Thus, for each of the materials, the ∆*K* levels at a specific, pre-defined crack growth rate *da*/*dN* for various chlorine contents were normalized to the corresponding ∆*K* level (i.e., equivalent *da*/*dN* value) for non-chlorinated water as a reference test condition. These normalized ∆*K* levels are plotted in Figure 9 as a function of the chlorine content indicating the data scatter covering a wider regime of crack growth rates. As pointed out above, in both materials, the most detrimental effect of water chlorination on the FCG resistance was seen for both materials from 0 ppm to 1 ppm free chlorine. However, while a further increase in chlorine content up to 10 ppm causes some further reduction in the FCG resistance of material PA-0, no such effect was found for the material PA-S1 containing the additional stabilizer package. In fact, the diagram also reveals that as soon as free chlorine in the water is present, the added stabilizer package in PA-S1 is beneficial to the FCG resistance.

## 4. Discussion

Regarding material failure in polymers by superimposed mechanical-environmental testing, two potential mechanisms in relation to the specific environment may play a role. On the one hand, the presence of an environmental medium may cause “physical aging” such as plasticization effects, post- and rec-crystallization or free volume changes in amorphous regimes. On the other hand, the combined action of high-local stresses and an aggressive environment (i.e., elevated temperature and environmental medium) may result in “chemical aging” locally at the crack tip, which leads to enhanced stabilizer consumption and rupture of covalent bonds of the polymeric molecules and thus ultimately to material degradation in the crack tip region [53,54,55,56,57,58,59,60,61,62,63,64,65]. This mechanism is facilitated by free volume increase and plastic deformation mechanisms in the highly stressed crack tip region, where a larger materials surface to volume ratio allows for a better interaction with the environmental medium [15]. The high oxidation-reduction potential (ORP) of chlorinated water accelerates the oxidation of polymeric materials, and therefore the deterioration of the material properties. Additional stabilizers may help to slow down the material degradation processes. A comparison of effect of chlorine content on the FCG resistances of the two formulations PA-0 and PA-S1 reveals the beneficial effect of the additional stabilization in PA-S1. While the added phenolic primary antioxidant acts as a radical scavenger and supports the material against the high ORP of free chlorine, the polymeric carbodiimide improves the hydrolysis stability. The combination of both stabilizers decelerates the material degradation in the specimen globally but also locally in the crack tip region. This effect is especially pronounced when comparing the relative change of FCG resistance in chlorinated water (10 ppm) to the FCG resistance in non-chlorinated water (0 ppm) of both materials.

## 5. Summary, Main Conclusions, and Outlook

To improve solar-thermal systems in terms of costs and weight, conventional materials like copper, aluminum or stainless steel are replaced by alternatives such as polyamides (PAs). In service, the materials utilized for solar-thermal system components are simultaneously exposed to mechanical loading and various environments (e.g., air, liquids, and elevated temperatures). To investigate the potential use of PAs for such solar-thermal systems applications, the effect of water with varying chlorine content on the failure behavior of two PA formulations differing in their stabilizer packages was investigated. A commercial PA reinforced with 30 wt % glass fiber containing a standard stabilization package (PA-0) was used as the reference material. For the other formulation, the reference material PA-0 was compounded with two additional stabilizers (PA-S1). To assess the long-term mechanical performance of these materials under near-service conditions, fatigue crack growth (FCG) experiments were performed with a unique test set-up allowing for superimposed mechanical-environmental loading of compact type (CT) specimens. The test temperature was 80 °C, reflecting a typical upper bound temperature of an integrated storage collector. Keeping the specimen configuration and geometry and the initial loading conditions the same, the total number of cycles to ultimate specimen failure was found to be reduced with an increase in chlorine content for both materials. As to the effect of the chlorine content on FCG kinetics, the most pronounced effect in enhancing the crack growth rates or decreasing the crack growth resistance was determined between 0 ppm and 1 ppm chlorine content, with some further deterioration of the FCG resistance with increasing chlorine content up to 10 ppm in one material (PA-0) and hardly any further effect in the other (PA-S1). When comparing the relative change of FCG resistance in chlorinated water (10 ppm) to the FCG resistance in non-chlorinated water (0 ppm), the additional stabilization in the material PA-S1 appears beneficial over the stabilization in the reference material PA-0. To further corroborate and generalize the above conclusion of a beneficial effect of the added stabilizer package in the glass fiber reinforced compound PA-S1, future investigations will be performed analogously with various unreinforced PA grades (PA6 and PA66) to eliminate any effects of an altered glass fiber concentration upon adding a stabilizer masterbatch.

## Figures and Tables

**Table 1:** Material designation and material composition.

Material Designation	Zytel 70F30HSL NC010	PA Supporting Material	Irganox 1098	Stabilizer 9000
PA-0	100 wt %	-	-	-
PA-S1	90 wt %	9 wt %	0.4 wt %	0.6 wt %
